# *Andrographis paniculata* restores gut health by suppressing inflammation and strengthening mucosal immunity

**DOI:** 10.3389/fphar.2025.1536683

**Published:** 2025-04-02

**Authors:** Vidushi Tyagi, Ashish Kumar, Karuna Shanker, Anirban Pal

**Affiliations:** ^1^ Bioprospection and Product Development Department, CSIR-CIMAP, Lucknow, India; ^2^ Academy of Scientific and Innovative Research (AcSIR), Ghaziabad, India; ^3^ Analytical Chemistry Department, Council of Scientific & Industrial Research-Central Institute of Medicinal and Aromatic Plants, Lucknow, India

**Keywords:** gut inflammation, intestinal immune system, cytokines, chemokines, *Andrographis paniculata*

## Abstract

**Background:**

Gut immunity plays a critical role in overall health by balancing tolerance to food antigens and microbiota while defending against pathogens. Inflammation and infection in the gut can disrupt this balance, leading to disease. *Andrographis paniculata*, a plant used in traditional medicine, is known for its anti-inflammatory and immune-modulating properties, making it a promising candidate for treating gut-related conditions.

**Methods:**

*A. paniculata* ethanolic extract (ApEtOH) was prepared by ethanol extraction of leaves, and bioactive compounds were identified using HPLC. Anti-inflammatory effects were evaluated *in vitro* using a Caco-2/RAW264.7 co-culture inflammation model via ELISA. Gene expression of chemokines in Caco-2 cells infected with *Salmonella* Typhimurium was assessed via quantitative real-time PCR. For *in vivo* studies, BALB/c mice were treated with ApEtOH at different doses, and the effects on bacterial load, immune response, and inflammation were assessed.

**Results:**

ApEtOH significantly downregulated the chemokines RANTES, MCP-1, and ENA-78, reducing the pro-inflammatory cytokines TNF-α and IL-6 *in vitro*. *In vivo* studies demonstrated reduced bacterial colonization in the spleen, lower systemic markers of infection, and restoration of intestinal homeostasis. ApEtOH normalized serum IgA, increased IgG, and decreased TNF-α and IL-10 levels. It also increased the expression of mucin (Muc-2) and lysozyme (Lyz-1), which are critical for epithelial integrity and antimicrobial defense.

**Conclusion:**

ApEtOH shows significant therapeutic potential for gut health by reducing bacterial colonization, modulating inflammation, and enhancing both innate and adaptive immunity. It may be a promising natural remedy for microbial induced gastrointestinal diseases and restoration of gut homeostasis.

## 1 Introduction

“Gut immunity” refers to the intricate immune ecosystem of the intestines that are tasked with maintaining tolerance to diverse food-derived antigens and commensal microbiota, while remaining vigilant and ready to defend against invading pathogens attempting to establish colonization in the gut. This balance or modulation of the responses is a critical factor in disease inception, which corroborates the fact stated by Hippocrates that “Almost all most diseases begin in the gut.” [SIC] Approximately 80% of the body’s immune system is housed in the gut lining, rendering the mammalian intestinal tract the body’s largest immune organ. It consists of both hematopoietic and non-hematopoietic cells, alongside trillions of microbes collectively known as the microbiota (Chassaing et al., 2014). While commonly referred to simply as “the gut,” the gastrointestinal tract is a complex system composed of three key components: a) the epithelial barrier layer, b) a diverse and formidable immune network that harbors the majority of the body’s immune cells, and c) the commensal microbiota (Clark and Coopersmith, 2007). Maintaining host health requires intestinal homeostasis, which is driven by the specialized mucosal immune system (MIS). Innate immunity defends against pathogens and activates adaptive responses by recognizing pathogen-associated molecular patterns (PAMPs), ensuring stable, precise immune regulation in the intestinal tract for effective protection and balance.

Toll-like receptors (TLRs, one of a kind), predominantly expressed in intestinal epithelial cells (IECs), play a key role in activating this response (Rebeca et al., 2011). In addition to acting as a physical barrier, the intestinal epithelium secretes a plethora of cytokines and chemokines that drive immune cell chemotaxis (Pott and Hornef, 2012). Upon bacterial invasion, IECs activate the mucosal inflammatory response by secreting epithelial neutrophil attractant-78 (ENA-78), IL-8, monocyte chemotactic protein-1 (MCP-1), and monocyte chemoattractant RANTES (regulated upon activation, normal T Cell expressed and secreted) (Yang et al., 1997). Significant amounts of antimicrobial peptides (AMPs) have been secreted by Paneth cells such as defensins, and lysozyme upon bacterial infection. These AMPs are crucial mediators of host-microbe interactions, playing a key role in maintaining homeostasis with colonizing microbiota and providing innate immune defense against enteric pathogens (Clevers and Bevins, 2013). The most prevalent adaptive immune component in the intestinal lumen is secretory IgA (sIgA), which plays a key role in maintaining intestinal homeostasis. It serves as a critical defense mechanism by inhibiting the adhesion and infiltration of pathogens across the intestinal barrier (Pietrzak et al., 2020).

According to a recent study, although mortality and disability-adjusted life years (DALYs) related to digestive diseases decreased significantly between 1990 and 2019, these conditions remain widespread. Pronounced disparities in the burden of digestive diseases persist among countries at varying levels of development (Wang et al., 2023). Thus, enacting policies for early screening and precision intervention could substantially alleviate the global burden of digestive diseases. This is where the role of herbal medicines comes in, which have been used for thousands of years for different conditions such as gastrointestinal health and disease prevention (Peterson et al., 2018). Modulation of the intestinal immune system by herbal medicines is a burgeoning frontier of research.

*Andrographis paniculata* (Burm. f.) Wall. ex Nees (*Ap*) commonly known as Kalmegh, belongs to the Acanthaceae family. It has been widely used in Ayurvedic, Chinese and Unani medicines for different disease conditions. The aerial parts of *Ap* are the most utilized, with their extracts containing diterpene glycosides, diterpenoids, flavonoids, lactones, and flavonoid glycosides (Hossain et al., 2014). A recent study revealed that administration of standardized *A. paniculata* extract increased lymphocyte counts and modulated cytokine production, indicating its ability to regulate immune function in healthy individuals (Rajanna et al., 2021). *Ap* attenuates methotrexate-induced intestinal and nephrotoxicity by alleviating inflammation via a decrease in TNF- α and IL-6 levels. It also helps in reducing oxidative stress and apoptosis (Parthasarathy and Prince, 2024). In intraabdominal sepsis, andrographolide attenuates the inflammatory immune response by promoting bacterial clearance, as it enhances macrophage phagocytic and bactericidal activities (Yu et al., 2023). In this study, we rigorously assessed the therapeutic potential of the ethanolic extract of *A. paniculata* (ApEtOH) in mitigating inflammation in the gut induced by pathogenic agents and thus strengthening the gut immune system.

## 2 Materials and methods

### 2.1 Preparation of the ethanolic extract of *Andrographis paniculata* (ApEtOH)

The aerial part (leaves) of *A. paniculata* (Accession no. CIMAP-939) was sourced from the experimental farm of CSIR-CIMAP, Lucknow. The leaves were shade-dried and coarsely powdered. The powder was then soaked in ethanol overnight and filtered (maceration method). The extract was then concentrated using a rotary evaporator (Buchi, Germany). It was then stored in aliquots at −20° until further analysis.

### 2.2 HPLC analysis of the ethanolic extract of *Andrographis paniculata*

Characterization and analysis for phytoconstituents of ApEtOH were done by High-performance Liquid chromatography (HPLC). For its preparation, the aerial part (leaves) was powdered and mixed with 95% alcohol (1:10 ratio). To analyze the extract, 10 mg of ApEtOH was taken and dissolved in 1 mL of HPLC-grade Methanol. A Shimadzu Nexera-XR system (Shimadzu, Kyoto, Japan) was used for HPLC analysis with an autosampler (SIL-20 AC XR), quaternary solvent delivery system (LC-20AD XR), online degasser (DGU-20A5R), PDA detector (SPD-M20A), and column oven (CTO-10ASVP). A C18 column (Phenomenex, Luna^®^, 250 × 4.6 mm, 5 μm) was used for chromatography in linear gradient mode. Solvent A- phosphate buffer and solvent B- Acetonitrile were used with an injection volume of 10 μL and a flow rate of 1.0 mL/min. Detection was done at 223 nm (Saxena et al., 2010).

**Table:** 

Time (min)	Flow rate (mL/min)	%B
0.01	1.0	5
18	1.0	45
25	1.0	80
30	1.0	80
35	1.0	5

### 2.3 Cell culture

RAW264.7 and Caco-2 cell lines were purchased from the National Centre for Cell Sciences (NCCS), Pune (India). RAW264.7 cells were maintained in DMEM (Dulbecco’s Modified Eagle’s Medium) with 10% FBS. Caco-2 cells were cultured in MEM (Modified Eagle’s Medium) with 20% FBS and 1% penicillin/streptomycin in a 37°C humidified incubator with 5% CO2.

### 2.4 Quantitative real-time PCR for chemokine estimation

Gene expression analysis for chemokines (MCP-1, ENA-78 and RANTES) was performed on Caco-2 cells. Briefly, Caco-2 cells were grown in 12-well plates at a density of 1 × 10^5^ cells per well. For 21 days, the media was changed on alternate days. After the monolayer was formed, cells were infected with a log phase of *Salmonella enterica* subsp. *enterica* serovar Typhimurium (*S*. Typhimurium) (MTCC 3232, India) at a bacterium-to-cell ratio/MOI of 100:1 for 1 h after centrifugation at 500 g for 5 min. Wells were then washed three times with 1X PBS (pH = 7.4) followed by gentamicin (100 μg/mL) treatment and incubated for 1 h (Luk et al., 2021). The wells were then washed three times with 1X PBS. The cells were treated with different concentrations (0.1, 1, 10 μg/mL) of ApEtOH and incubated for 6 h. Washing was performed as described above and total RNA was extracted from the cells using TRIzol (Invitrogen™) according to the manufacturer’s protocol. The amount of RNA was assessed using the 260/280 absorbance ratio measured by a NanoDrop Lite (Thermo Fisher Scientific, United States). For cDNA synthesis, 1 μg of total RNA was used according to the manufacturer’s instructions with the high-capacity cDNA Reverse Transcriptase Kit (Invitrogen, United States). Real-time PCR was performed in a 10 μL reaction volume containing 1 μg cDNA, 0.5 μmol/L of each primer, and PowerUp™ SYBR™ Green Master Mix (2X). Cycling conditions included an initial 95°C for 15 min, followed by 40 cycles of 95°C for 15 s, 60°C for 15 s, and 72°C for 10 s. Reactions were performed on a QuantStudio™ Real-Time PCR System (Thermo Fisher Scientific). Gene expression was normalized to a housekeeping gene (GAPDH), and relative gene expression changes were analyzed using the 2^−ΔΔCT^ method. Human-specific primer sequences used in qPCR of RANTES, MCP-1, ENA-78 and GAPDH (as a housekeeping gene) (listed in Supplemantary Table 1).

### 2.5 *In vitro* intestinal inflammation co-culture model

**FIGURE 1:**
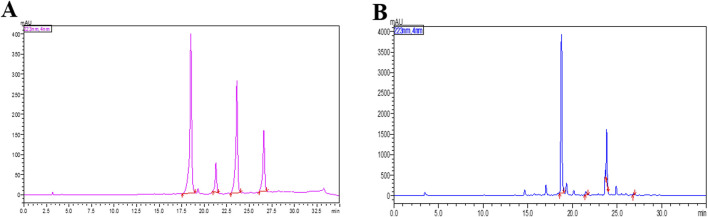
HPLC chromatogram at 223 nm of **(A)** The Standard mix **(B)** The Ethanolic extract of *Andrographis paniculata* (ApEtOH) containing Andrographolide- 12.43%, Neo-andrographolide- 1.32%, 14-DDA- 5.25%, Andrograpanin- 0.24%.

Caco-2 cells were seeded at a density of 1 × 10^5^ cells per well in the apical chamber (AP) of Polyethylene Terephthalate (PET) inserts of 12-well transwell plates (pore size; 0.4 μm). The culture medium was refreshed every 2–3 days for a total of 21 days. Their differentiation status was monitored every third day using an EVOM2 Volt-ohmmeter (WPI, United States) to measure Transepithelial Electrical Resistance (TEER), a direct indicator of Caco-2 monolayer integrity and barrier function (Supplemantary Figure 1). Meanwhile, prior to Caco-2 complete differentiation, RAW 264.7 cells were seeded into the basolateral chambers (BL) at a density of 1 × 10^5^ cells per well and incubated for 48 h at 37°C in a 5% CO_2_ atmosphere to allow for cell adhesion. After this period, all media were replaced with 10% FBS DMEM. LPS (1 μg/mL) was added to the BL chamber of RAW264.7 cells. Treatment was given with different concentrations (0.01, 0.1, 1, 10 μg/mL) of ApEtOH in the AP chamber for 12–18 h. Cell supernatant was collected from AP and pro-inflammatory cytokines (TNF-α and IL-6) were quantified using human ELISA kits (BD Biosciences), according to the manufacturer’s instructions (Kim et al., 2021; Yang et al., 2022).

### 2.6 *In vivo* experiment

Female BALB/c mice (22–25 g) were randomly divided into six groups. The animals were bred and maintained in-house at a temperature of 25°C ± 2°C with humidity control, and subjected to a 12-h light/dark cycle. They were fed a standard diet (Altromin, Germany) and drinking water *ad libitum*. Each group consisted of six mice (n = 6). To study the systemic infection, inflammation and effect on immunity, BALB/c mice were fed orally with the vehicle control (0.6% Carboxy Methyl Cellulose), different concentrations of ApEtOH (250, 500 and 750 mg kg^−1^ body weight) and a positive control as a commercial probiotic (VSL#3, rich in *Lactobacilli* and *Bifidobacterium*) for 28 days (Supplemantary Table 2). On Day 14, mice were immunized with 10^3^ CFU *S*. Typhimurium via the intraperitoneal route. On Day 28, mice were challenged with log-phase cultures of bacteria (pelleted and re-suspended in sterile PBS buffer, 1 × 10^9^ CFU) after neutralization of gastric acid with 5% Sodium Bicarbonate. Mice were euthanized on day fourth of the challenge infection. All animal experiments were approved by the Institutional Animal Ethics Committee of CSIR-CIMAP, Lucknow, India under the aegis of the Committee for the Purpose of Control and Supervision of Experiments on Animals (CPCSEA), protocol (CIMAP/IAEC/2023-26/5).

#### 2.6.1 RNA extraction and quantitative real-time PCR

Total RNA was isolated from the mouse intestine using TRIzol (Invitrogen™). cDNA synthesis and RT-PCR were performed as described in Section 2.4. Mouse-specific primer sequences for TNF-α, Lysozyme-1 (Lyz-1), mucin (Muc-2) and GAPDH were used (listed in Supplemantary Table 1).

#### 2.6.2 Bacterial colonization

The reticulo-endothelial organ (spleen) was weighed, homogenized, and macerated to prepare a single-cell suspension in sterile 1X PBS. Dilutions were spread on nutrient agar plates and incubated overnight for bacterial growth. Colony-forming units (CFUs) were counted and CFUs per gram of spleen were quantified.

#### 2.6.3 IgG and serum IgA quantification

Blood was collected from the retro-orbital plexus and serum was isolated for antibody (IgG and IgA) titer. Serum IgG antibodies specific for *S*. Typhimurium (MTCC 3232) were detected by ELISA. Briefly, 96-well plates (Maxisorp; Nunc) were coated with 100 μL/well of *S*. Typhimurium protein (1.25 μg/mL) diluted in carbonate buffer (pH 9.0) overnight at 4°C. The plate was washed three times with wash buffer (0.05% Tween-20 in 1X PBS) and blocked with assay diluent (10% FBS in 1X PBS) for 1 h at room temperature. Washing was performed as previously described and serum samples were added at appropriate dilutions (in 10% FBS in 1X PBS) and incubated for 2 h at room temperature. After washing (5 times), the detection antibody (horseradish peroxidase-conjugated goat anti-mouse IgG antibody; Thermo Fisher Scientific, United States) was added and incubated for 1 h. After incubation, 3,3′,5,5′-Tetramethylbenzidine substrate (Thermo fischer Scientific) and hydrogen peroxide were added and incubated for 30 min. The reaction was then stopped by adding 2N sulfuric acid. The absorbance was measured at 450 nm. The antibody titers were expressed as the reciprocal of the highest dilution with an absorbance greater than that of the control well to which serum from untreated mice was added. IgA was also quantified using an ELISA kit (Invitrogen) according to the manufacturer’s protocol.

#### 2.6.4 Quantification of *in vivo* cytokines

ELISA was performed for the quantification of cytokines (TNF-α and IL-10) from serum samples according to the manufacturer’s protocol using mouse ELISA kits (BD Biosciences).

### 2.7 Statistical analysis

All groups were compared by one-way analysis of variance (ANOVA), and the significance of the mean differences between groups was determined by Dunnett’s multiple comparison test. Results are presented as mean ± SD (standard deviation). Statistical analysis was conducted using GraphPad Prism software. A two-tailed probability (α = 0.05) was considered statistically significant, with p < 0.05 represented as *, p < 0.01 as ** and p < 0.001 as ***.

## 3 Results

### 3.1 HPLC profile of ethanolic extract of *Andrographis paniculata* (ApEtOH)

Figure 1 shows the HPLC fingerprint of (A) The Standard mix and (B) ApEtOH. The content of marker compounds detected in ApEtOH was Andrographolide- 12.43%, Neo-andrographolide- 1.32%, 14-DDA- 5.25%, Andrograpanin- 0.24%.

### 3.2 Effect of *Andrographis paniculata* ethanolic extract on chemokine gene expression

**FIGURE 2:**
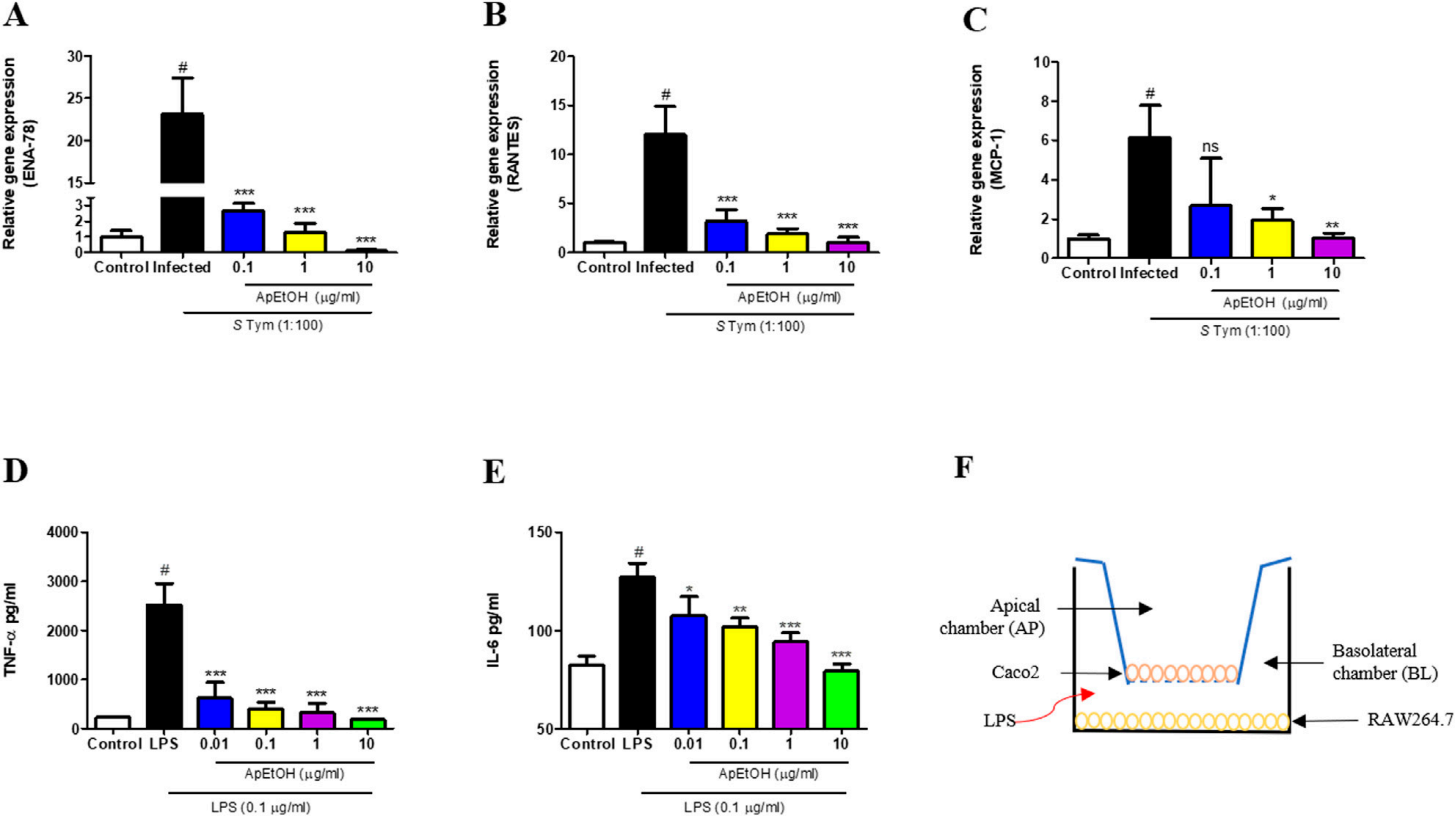
Effect of ApEtOH on chemokine gene expression and pro-inflammatory cytokine release. **(A–C)** Gene expression analysis of ENA-78, RANTES and MCP-1 respectively in control, infected (*S*. Typhimurium, MOI 1:100) and ApEtOH treated (0.1, 1 and 10 μg/mL) Caco-2 cells. TNF-α **(D)** and IL-6 **(E)** quantification from cell supernatant of control, LPS stimulated and ApEtOH treated (0.01, 0.1, 1 and 10 μg/mL) co-culture of Caco-2/RAW264.7. **(F)** Represents Caco-2/RAW264.7 co-culture model in transwell plate. Data are presented as mean ± SD (*n* = 3), #*p* < 0.001 versus the control, *p < 0.05, **p < 0.01, ***p < 0.001 and ns (not significant) versus the infected group and the LPS group alone respectively.

The *in vitro* anti-inflammatory activity of ApEtOH was assessed using the intestinal cell line Caco-2. Upon stimulation with the pathogen (*Salmonella* Typhimurium), Caco-2 cells initiated an inflammatory response characterized by the secretion of chemokines, which have a pivotal role in orchestrating acute inflammation. Notably, ApEtOH, at concentrations of 0.1, 1, and 10 μg/mL, exhibited a profound inhibitory effect, significantly reducing the expression of C-X-C chemokine (ENA-78) and C-C chemokine (RANTES) compared to the infected group alone (Figures 2A, B). However, for MCP-1 expression, only 1 and 10 μg/mL of ApEtOH were found to be significant while 0.1 μg/mL did not show any significant difference when compared with the infected group (Figure 2C). This suppression underscores the significant anti-inflammatory potential of ApEtOH, highlighting its ability to effectively modulate critical inflammatory mediators.

### 3.3 Evaluation of the anti-inflammatory effects of ApEtOH in an *In vitro* model of gut inflammation

To assess the impact of ApEtOH on gut inflammation, an *in vitro* study was conducted using a co-culture model of Caco-2 and RAW 264.7 cells, designed to simulate intestinal inflammatory conditions. In this model, RAW 264.7 macrophages were stimulated with LPS, leading to the release of pro-inflammatory cytokines, such as TNF-α and IL-6. These cytokines triggered a cytokine storm that exacerbated inflammation in Caco-2 cells, mimicking the pathogenesis of intestinal inflammation. Treatment with ApEtOH at concentrations of 0.1, 1, and 10 μg/mL led to a substantial reduction in the secretion of key pro-inflammatory cytokines, such as TNF-α and IL-6 (Figures 2D, E). This pronounced inhibition of cytokine production underscores the potent anti-inflammatory properties of ApEtOH, indicating its potential as a therapeutic agent for the management of various intestinal inflammatory conditions by mitigating excessive immune responses and cytokine-mediated damage. Figure 2F shows the *In vitro* Caco-2/RAW264.7 co-culture model in a transwell plate.

### 3.4 *In vivo* experiment

#### 3.4.1 Effect of ApEtOH on TNF-α, lysozyme and mucin gene expression

**FIGURE 3:**
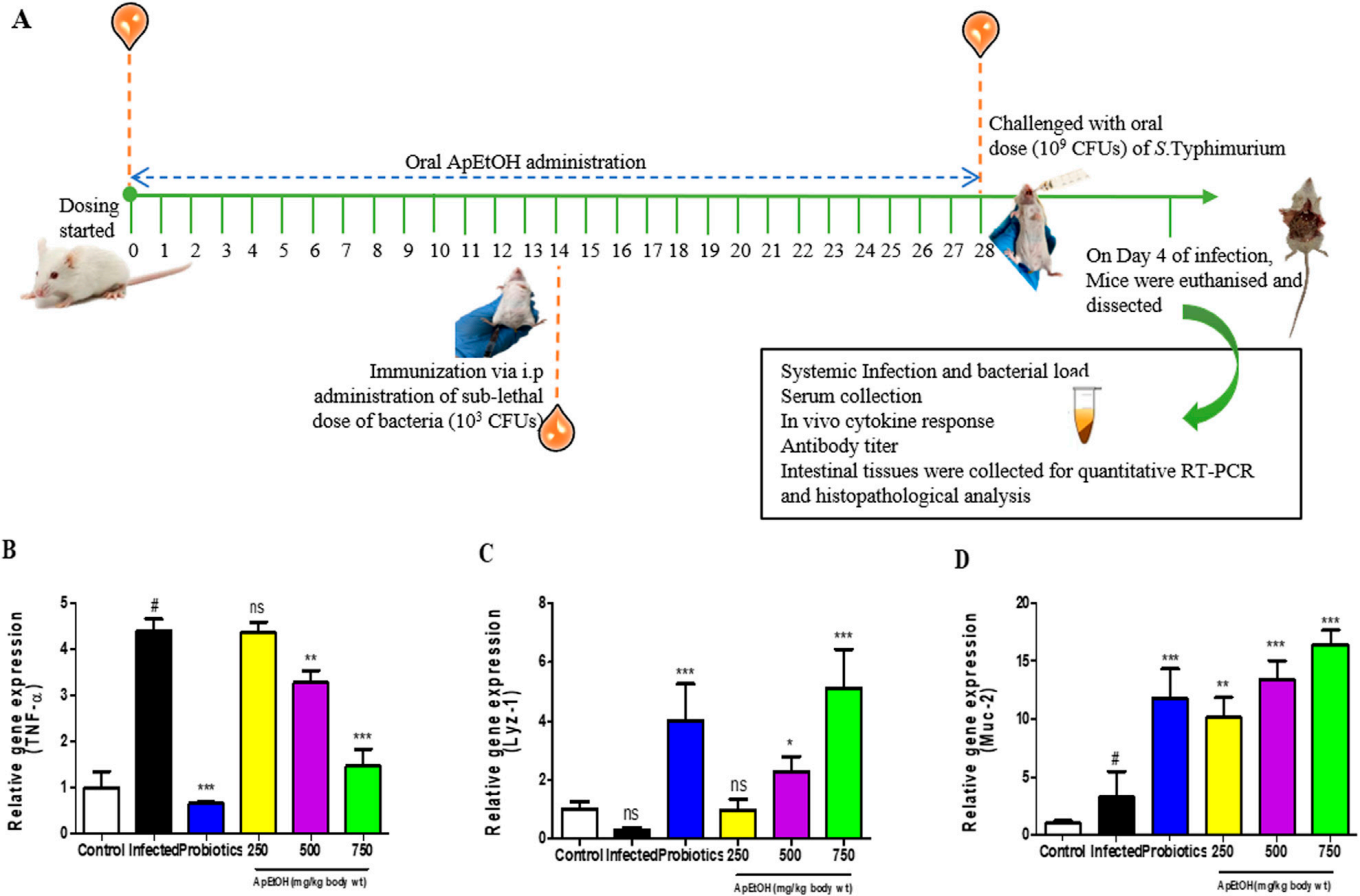
Effect of ApEtOH on animal studies. **(A)** is a schematic overview of the *in vivo* experimental approach. **(B–D)** TNF-α, Lyz-1 and Muc-2 gene expression analysis in control, infected and ApEtOH-treated groups (250, 500 and 750 mg/kg bdwt). Data are presented as mean ± SD (*n* = 6), #*p* < 0.05 versus the control in panel D, *p < 0.05, **p < 0.01, ***p < 0.001 and ns (not significant) versus the infected group.

Figure 3A provides a schematic overview of the *in vivo* experimental approach. Quantitative RT-PCR results from intestinal tissues show significant downregulation of TNF-α in 500 and 750 mg/kg bdwt ApEtOH treated groups compared to the infected group, demonstrating its anti-inflammatory potential (Figure 3B). The study assessed the effects of ApEtOH on innate immune responses, focusing on AMPs (Lyz-1) and Muc-2 gene expression. ApEtOH treatment (500 and 750 mg/kg bdwt) markedly increased Lyz-1 expression (Figure 3C), which may mitigate bacterial translocation and infection progression. Similarly, Muc-2, a key mucus component secreted by epithelial cells, was significantly upregulated by ApEtOH (Figure 3D), which could enhance the protective mucus barrier, and prevent pathogen translocation. This suggests that ApEtOH may strengthen mucosal immunity by strengthening both physical and antimicrobial defenses.

#### 3.4.2 Bacterial colonization

**FIGURE 4:**
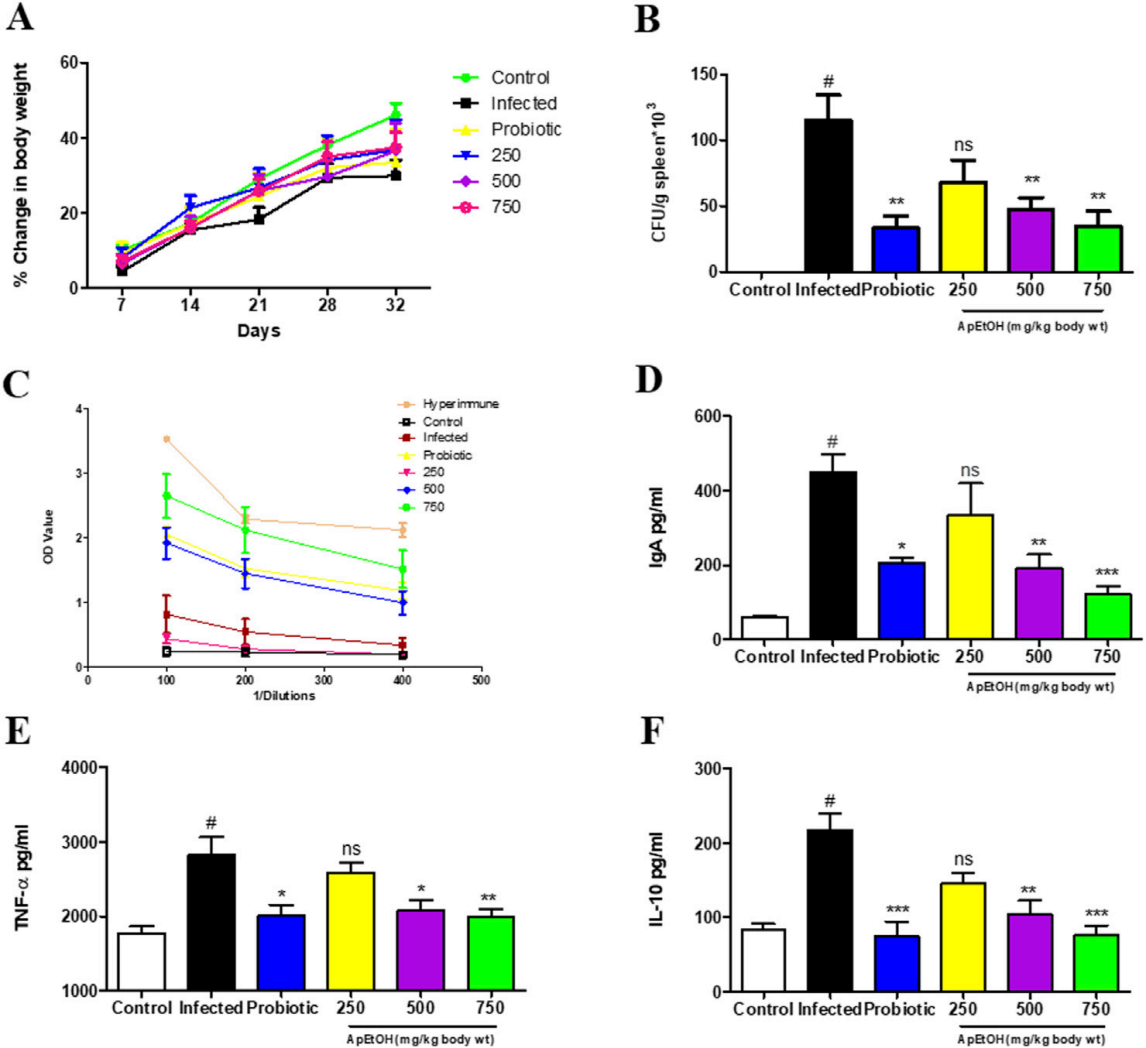
Immunological behavior of ApEtOH in *in vivo* studies. **(A)** Percentage change in body weight **(B)** Bacterial colonization expressed as CFU/g spleen*10^3^. **(C–F)** ELISA was performed for IgG, serum IgA, TNF-α and IL-10 quantification respectively from the serum samples of control, infected and ApEtOH-treated groups (250, 500 and 750 mg/kg body wt). Data are presented as mean ± SD (*n* = 6), #*p* < 0.001 versus the control, *p < 0.05, **p < 0.01, ***p < 0.001 and ns (not significant) versus the infected group.

Microbial load analysis in the spleen was conducted on day 4 post-infection. While there was no statistically significant difference in the percentage change in body weight (Figure 4A), a marked increase in microbial load was observed in the infected group. However, mice treated with the probiotic and ApEtOH at doses of 500 and 750 mg/kg bdwt showed a significant reduction in bacterial colonization, as evidenced by the notable decrease in microbial load in the spleen compared to the infected group alone (Figure 4B). In contrast, the 250 mg/kg bw ApEtOH-treated group did not show a significant reduction in microbial load when compared to the infected group.

#### 3.4.3 Effect of ApEtOH on humoral immunity

The humoral immune response was evaluated by measuring *Salmonella*-specific IgG and IgA serum levels in various treatment groups. Notably, mice treated with 750 mg/kg bw ApEtOH exhibited a significant increase in IgG titers, as shown in Figure 4C, where the reciprocal of the highest dilution showed an absorbance higher than that of the infected group. A hyperimmune serum previously raised in the same host species was used as a positive control, as it is expected to contain high titers of *Salmonella*-specific IgG. However, no significant differences were observed in the IgG levels of the groups treated with 250 and 500 mg/kg bw ApEtOH. Interestingly, serum IgA levels were elevated in the infected mice compared to the normal mice, suggesting that IgA plays a critical role in the inflammatory response. IgA may contribute to acute inflammation during bacterial invasion as part of the body’s defense mechanism to eliminate the pathogen. However, treatment with ApEtOH at doses of 500 and 750 mg/kg bw significantly reduced serum IgA levels compared to the infected group, indicating reduced inflammation (Figure 4D).

#### 3.4.4 *In vivo* cytokine response

Furthermore, ApEtOH at these doses (500 and 750 mg/kg bw) was found to mitigate inflammation induced by bacterial invasion, as evidenced by the significant decrease in the secretion of the pro-inflammatory cytokine TNF-α when compared to the infected group. IL-10, a cytokine required by *S.* Typhimurium systemic infection in mice, was also significantly reduced in the 500 and 750 mg/kg bw ApEtOH-treated groups (Figures 4E, F), highlighting the role of ApEtOH in suppressing systemic infection and controlling inflammation. Taken together, these data suggest that ApEtOH, particularly at doses of 500 and 750 mg/kg bw, not only reduces bacterial colonization but also modulates the immune response, reduces inflammation and effectively curbs systemic infection.

## 4 Discussion

The mammalian gut immune system integrates innate and adaptive elements to defend against pathogens and maintain homeostasis. Disruptions can weaken immunity, increasing susceptibility to infections and autoimmune conditions. Balanced regulation is crucial for health, leading to growing interest in restoring gut immunity to prevent disease and promote overall wellbeing (Zheng et al., 2020). Herbal medicine is gaining global recognition for its therapeutic potential and minimal side effects. Its holistic benefits make it a preferred alternative to pharmaceuticals (Ding et al., 2024). Moreover, the existing literature is insufficient to provide robust scientific validation of herbal plant extracts for their potential to restore gut immunity.

Therefore, in the present study, we evaluated the therapeutic potential of ApEtOH in mitigating gut inflammation triggered by pathogenic infection, providing valuable insights into its possible application as a treatment for pathogen-induced intestinal conditions. ApEtOH was prepared from the leaves of *Andrographis paniculata*. HPLC profiling exhibits the presence of some marker compounds such as Andrographolide, Neo-andrographolide, 14-DDA, and Andrograpanin. The effect of ApEtOH on the intestinal immune system was investigated through *in vitro* and *in vivo* studies.

Toward the innate immune response (first line of defense), the effect on Muc-2 and AMP (Lyz-1) gene expression analysis was assessed via *in vivo* studies. Foreign antigens were eliminated by physical barriers such as mucus and chemical barriers such as AMPs on the epithelial layer, preventing pathogen entry. The mucus, secreted by epithelial cells, serves as the first line of physical defense in the intestines. It acts as a protective barrier between the mucosa and the luminal contents (Atuma et al., 2001). ApEtOH was found to significantly upregulate the expression of Muc-2, a large glycoprotein and principal constituent of mucus. This upregulation likely plays a pivotal role in inhibiting pathogen translocation, thereby strengthening the mucosal immune system and enhancing the body’s protective barriers against microbial invasion. AMPs, critical components of the host defense system, play a vital role in safeguarding the host from pathogenic threats (Moretta et al., 2021). Lysozyme, a potent AMP, exhibits robust antimicrobial activity. However, intestinal invasion by enteric pathogens, such as *S.* Typhimurium, significantly suppresses lysozyme expression, exacerbating infection and inflammation (Salzman et al., 2003). Remarkably, treatment with ApEtOH (500 and 750 mg kg^−1^ body weight) markedly upregulated the expression of Lyz-1 compared to the infected group. This enhanced expression may effectively limit bacterial translocation to surrounding tissues, thereby mitigating the progression of infection.

Focusing on adaptive immunity, the humoral immune response was evaluated by assessing *Salmonella*-specific IgG and serum IgA. Concurrently, the impact on cell-mediated immunity was examined through the analysis of pro-inflammatory cytokine release and chemokine gene expression. It was observed that ApEtOH significantly downregulated the expression of C-C chemokines, such as RANTES and MCP-1, along with C-X-C chemokines such as ENA-78 in response to bacterial invasion. The C-X-C chemokines serve as powerful chemoattractants for neutrophils, while the C-C chemokines play a pivotal role in the efficient recruiting of monocytes during infection (Baggiolini et al., 1993). The secretion of these chemokines in response to infection triggers the onset of an acute mucosal inflammatory response, a key pro-inflammatory function of intestinal epithelial cells (Yang et al., 1997), which, if not regulated results in detrimental conditions. Additionally, certain C-C and C-X-C chemokines have been shown to attract T cells, thereby establishing a potential connection between inflammatory responses and activation of the immune system (Qin et al., 1996). The initiation of this robust inflammatory cascade was markedly suppressed by ApEtOH.

Furthermore, the effect of ApEtOH on cytokine release in intestinal inflammation was studied on an *in vitro* gut inflammation model (Caco-2/RAW 264.7 co-culture model), which simulated the intestinal inflammatory system. This further substantiated our findings (Supplemantary Figure 3), reinforcing that ApEtOH has strong anti-inflammatory properties. These findings were supported by *in vivo* studies where ApEtOH was found to significantly reduce serum TNF-α release along with its gene expression in intestinal tissue. Its ability to reduce the Th1 response, as evidenced by the reduction of the key pro-inflammatory cytokines TNF-α and IL-6, underscored its role in mitigating immune-driven inflammation. However, the mechanism of anti-inflammatory action was explored in this study and available literature suggests that marker compounds (Andrographolide, Neo-andrographolide, 14-DDA, and Andrograpanin) present in ApEtOH exert anti-inflammatory properties via inhibition of NF- κB activation (Xia et al., 2004; Yin et al., 2022; Chao et al., 2010).

IL-10 production is critical for facilitating *S*. Typhimurium infection, aiding the bacterium in establishing systemic dissemination by suppressing the host immune response (Salazar et al., 2017). Notably, ApEtOH treatment significantly reduced serum IL-10 levels, effectively limiting systemic infection. This aligns with our observation of a substantial reduction in microbial load within the reticuloendothelial organ (spleen), highlighting its therapeutic potential in controlling bacterial spread.

Treatment with ApEtOH led to a significant elevation in *Salmonella*-specific IgG antibody titers, demonstrating a strengthening in humoral immunity. *Salmonella*’s ability to breach the intestinal epithelial barrier triggered an IgA-mediated immune response that amplified inflammation (Chang et al., 2014). ApEtOH treatment effectively suppressed excessive serum IgA levels, indicating a significant reduction in inflammation and the restoration of gut homeostasis. However further investigation is needed to elucidate a more comprehensive mechanism. Moreover, ApEtOH was found to be non-toxic in acute and sub-acute *in vivo* oral toxicity studies (Supplemantary Tables 3, 4; Supplemantary Figures 5, 6).

By assessing the effects of ApEtOH on immunity and inflammation, this study advances our understanding of the role of ApEtOH in restoring gut health under conditions of microbial-induced inflammation. Our investigation provides crucial insights about ApEtOH, which exerts its anti-inflammatory effects, thereby enhancing our understanding of its role in alleviating gut inflammation associated with microbial infection. This exploration not only highlights the efficacy of ApEtOH as a potential natural remedy but also contributes to the broader knowledge of herbal interventions in the management of inflammation-driven gastrointestinal disorders.

## 5 Conclusion

These findings highlight the potential of *A. paniculata* ethanolic extract (ApEtOH) as a natural therapeutic agent for microbial-induced gut inflammation. By strengthening mucosal defense, modulating immune responses, and suppressing pro-inflammatory mediators, ApEtOH demonstrates significant immunomodulatory and anti-inflammatory properties. While the precise mechanism of action requires further investigation, the inhibition of NF-κB activation by its bioactive compounds suggests a promising pathway for therapeutic intervention.

## Data Availability

The datasets presented in this study can be found in online repositories. The names of the repository/repositories and accession number(s) can be found in the article/Supplemantary Material.
