# Correction: *Andrographis paniculata* restores gut health by suppressing inflammation and strengthening mucosal immunity

**DOI:** 10.3389/fphar.2025.1637737

**Published:** 2025-06-17

**Authors:** Vidushi Tyagi, Ashish Kumar, Karuna Shanker, Anirban Pal

**Affiliations:** ^1^ Bioprospection and Product Development Department, CSIR-CIMAP, Lucknow, India; ^2^ Academy of Scientific and Innovative Research (AcSIR), Ghaziabad, India; ^3^ Analytical Chemistry Department, Council of Scientific & Industrial Research-Central Institute of Medicinal and Aromatic Plants, Lucknow, India

**Keywords:** gut inflammation, intestinal immune system, cytokines, chemokines, *Andrographis paniculata*

In the published article, there was an error regarding the **Affiliation** for Anirban Pal. As well as having affiliation 1, they should also have affiliation 2 Academy of Scientific and Innovative Research (AcSIR), Ghaziabad, India.

The authors apologize for this error and state that this does not change the scientific conclusions of the article in any way. The original article has been updated.

