# Supplementary material for: Andrographis paniculata restores gut health by suppressing inflammation and strengthening mucosal immunity
Source: Front Pharmacol. 2025 Apr 2;16:1536683. doi: 10.3389/fphar.2025.1536683 (PMC12000883; doi:10.3389/fphar.2025.1536683)
Supplement: Supplementary file 1 [file DataSheet1.pdf]

Supplementary data:

Caco-2 cells were cultured in apical chamber inserts to form differentiated monolayers. Their differentiation status was monitored every third day using an EVOM2 Volt-ohmmeter (WPI, USA) to measure Transepithelial Electrical Resistance (TEER). TEER may serve as a direct indicator of the structural integrity and barrier function of Caco-2 cell monolayers.

TEER was calculated as:

Actual TEER = (TEER of cell-bearing insert−TEER of blank insert) × insert area (1.12 cm^2)

Usually when the TEER values >400 $\Omega \cdot cm^2$ on the apical side of the Transwell plate, the cells were expected to exhibit dense confluent monolayer, that may be indicative of optimal barrier integrity and functionality. Monolayers with TEER values >400 $\Omega \cdot cm^2$ were deemed suitable for the in vitro studies (figure 1).

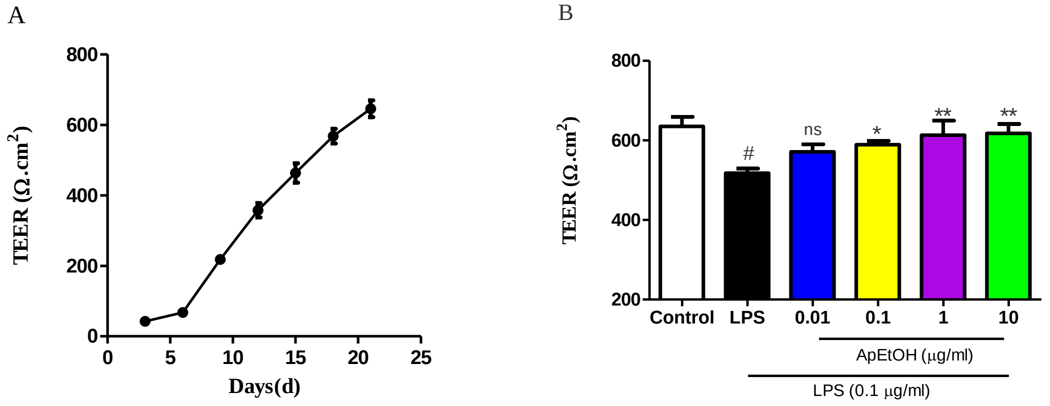

Figure 1: Represents TEER measurements of Caco-2 cells over time (n = 3 readings per day) whereas figure B) represents TEER values after 12 hr of LPS treatment in coculture model. Data presented as mean ± SD (n = 3) #p < 0.05 versus the control group, *p < 0.05 and **p < 0.01 and non-significant (ns) with respect to LPS alone.

Table1:

Gene		Forward (5'-3')	Reverse (5'-3')	Accession number
Human	RANTES	CTTGAAGGGCCCAGATTCTAC	TCCCAAAGTGCTGGGATTAC	NM_002985
	MCP-1	AGTCTCTGCCGCCCTTCT	GTGACTGGGGCATTGATTG	NM_002982
	ENA-78	CAATCTTCGCTCCTCCAATCT	TTTACAGACCACGCAAGGAG	NM_002994
	GAPDH	GACCACTTTGTCAAGCTCATTTC	CTCTCTTCCTCTTGTGCTCTTG	NM_002046
Mouse	TNF-α	ACGGCATGGATCTCAAAGAC	GTGGGTGAGGAGCACGTAGT	NM_013693
	LYZ-1	GAGACCGAAGCACCGACTATG	CGGTTTTGACATTGTGTTCGC	NM_013590
	MUC-2	TGCCCAGAGAGTTTGGAGAGC	CCTCACATGTGGTCTGGTTG	NM_023566
	GAPDH	TACACTGAGGACCAGGTTGT	CTGTAGCCGTATTCATTGTC	NM_008084

Table2:

Groups	Treatment
1	Normal mice
2	Infected mice (*S.* Typhimurium)
3	Infection + Probiotics
4	Infection +ApEtOH treated 250 mg kg^{-1} body weight
5	Infection +ApEtOH treated 500 mg kg^{-1} body weight
6	Infection +ApEtOH treated 750 mg kg^{-1} body weight

Cell viability: For *in vitro*, cell viability assay (MTT assay) was performed on both RAW264.7 and Caco2 cell line. 5×10^5 cells/ml were seeded in 96 well plate in 37°C humidified incubator with 5% CO2. Cells were then treated with control, different concentrations of ApEtOH for 12-18h. 100µl/well MTT reagent (1mg/ml) was then added and incubated for 2-4hr. Plate was observed under microscope for the formation of formazan crystals. 100µl Solubilization solution (DMSO) was added to each well. Incubated for 30min for dissolution of crystals. Absorbance was measured at 570nm.

Result: There was no significant effect on viability of RAW264.7 cells on tested concentrations of ApEtOH while it was found to be safe till 100 µg/ml in Caco2 cells.

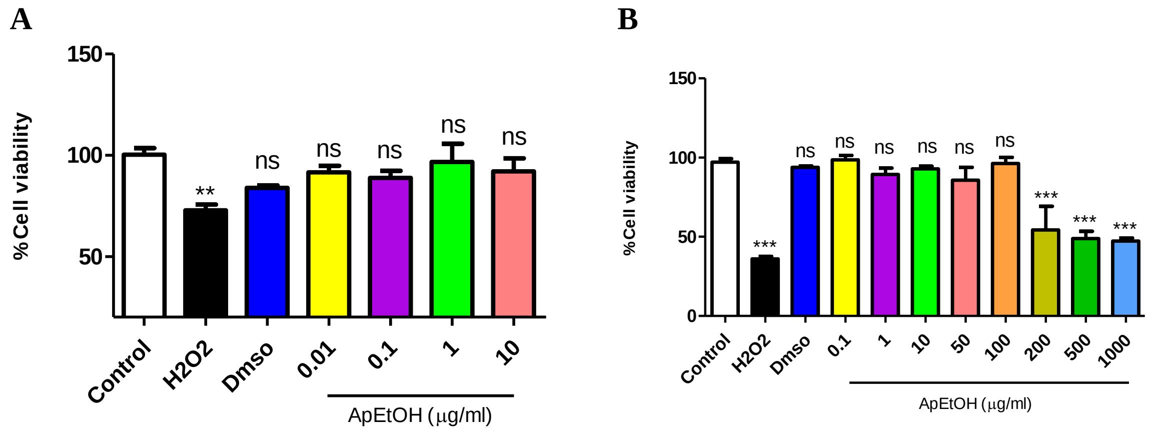

FIGURE 2: Effect of ApEtOH on cell viability of (A) RAW264.7 and (B) Caco2 cells. Dmso was taken as vehicle control and H_2O_2 as positive control. Data presented as mean ± SD (n = 3), $**p < 0.01$, $***p < 0.001$ and ns (non-significant) with respect to the control group.

Quantification of cytokines: Assay was performed on RAW264.7 cell line. 1×10^5 cells were seeded in 96 well plate overnight in 37°C humidified incubator with 5% CO2. Cells were then treated with control, LPS (1µg/ml), different concentrations (0.01, 0.1, 1, 10µg/ml) of extract for 12-18h. Cell supernatant were collected, stored at -20°C. Cytokines (TNF-α and IL-6) in culture supernatants were quantified by mouse ELISA kits (BD Biosciences), following the manufacturer instructions.

Result: TNF-α and IL-6 quantification from cell supernatant treated with ApEtOH shows significant decrease when compared with LPS alone.

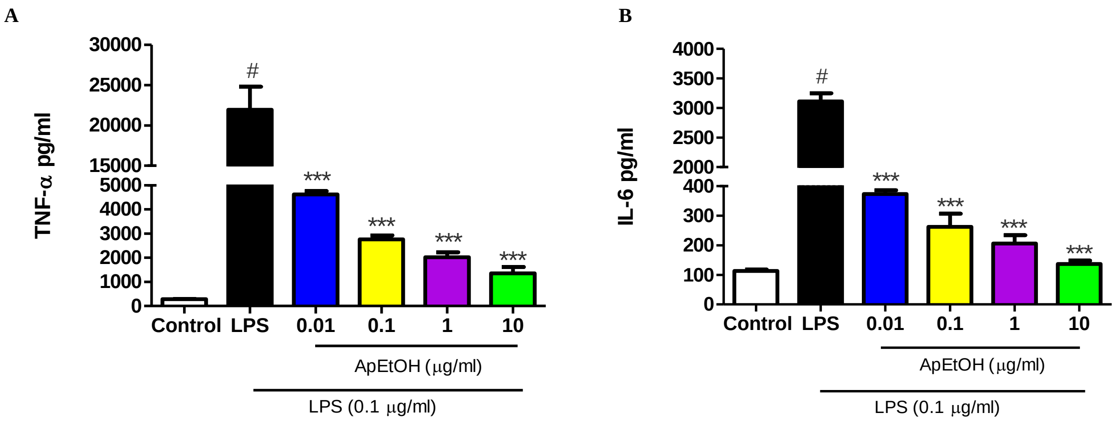

FIGURE 3: Effect of ApEtOH on TNF-α and IL-6 release in LPS stimulated RAW264.7 cells. Data presented as mean ± SD ($n = 3$), #$p < 0.001$ versus the control group, *$p < 0.05$, **$p < 0.01$, ***$p < 0.001$ with respect LPS alone only.

Histopathology: Part of small intestine (ileum) of mice has been removed and washed with 1X PBS. Small sections were cut using microtome. Tissue sections were air dried. Sections were then stained with filtered 0.1% Mayers Haematoxylin for 10 minutes in a 50 ml conical tube. Sections were rinsed in cool running double distilled water for 5 minutes. It was then dipped in 0.5% Eosin for 12 times. Sections were then put in distilled water until eosin stops streaking. Slides were first dipped in 50% ethanol and then in 70% ethanol 10 times each. Equilibrate in 95% EtOH for 30 seconds and then in 100 % ethanol for 1 minute. Dipped in xylene for several times. Slides were then cleaned and mounted with DPX (Dibutylphthalate Polystyrene Xylene) and observed under microscope.

Result: Histopathological analysis revealed severe disruption in intestinal architecture within the infected group, characterized by pronounced crypt and villi distortion, goblet cell depletion, structural disorganization of smooth muscle layers, and extensive infiltration of inflammatory cells. In contrast, significant restoration of villi and crypt integrity was observed in groups treated with probiotics and ApEtOH. Notably, the 250 mg/kg bd wt dose of ApEtOH showed minimal improvement, demonstrating negligible effects on intestinal recovery compared to the infected group, corroborating its limited efficacy at this dosage. However, treatment with 500 and 750 mg/kg bd wt of ApEtOH led to substantial restoration of intestinal morphology, with marked recovery of crypt and villi structure, indicative of its robust protective and reparative properties. These findings underscore the potent therapeutic potential of ApEtOH at higher doses in mitigating pathogen-induced intestinal damage.

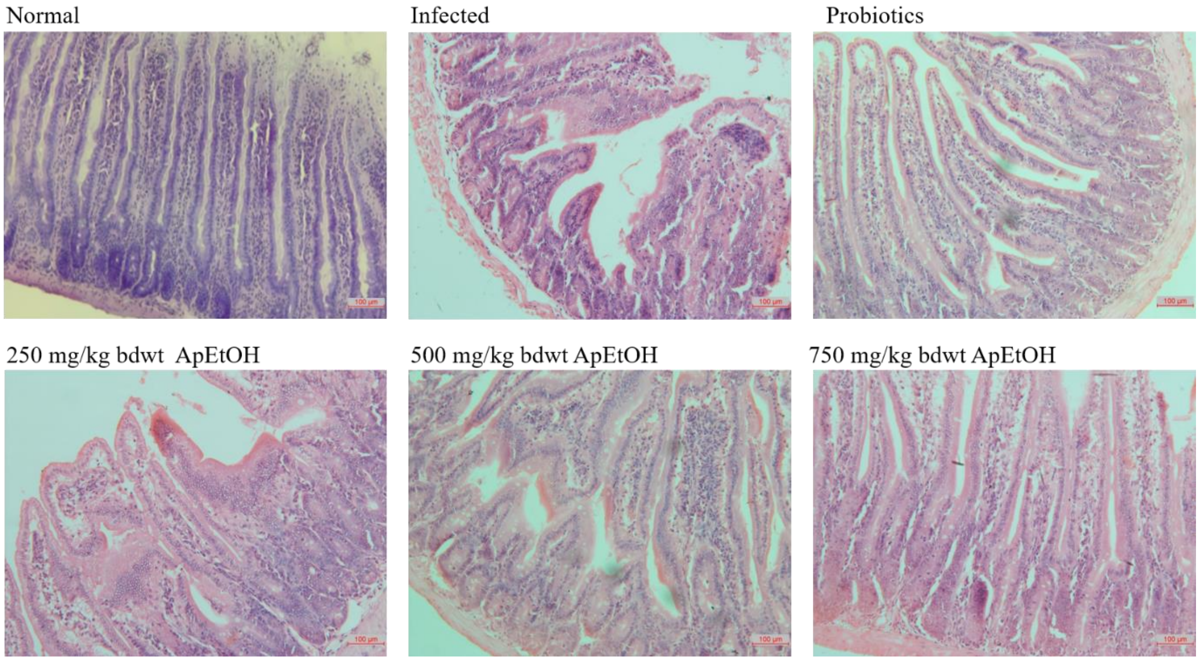

FIGURE 4: Hematoxylin and Eosin (H&E) staining images of mouse intestines, showing sections from the control group (untreated and uninfected), the infected group (*S. Typhimurium*-infected), and groups treated with probiotics or ApEtOH at varying doses (250, 500, and 750 mg/kg body weight).

Safety studies:

Acute Toxicity of ApEtOH: Acute oral toxicity of ApEtOH was conducted according to the Organization for Economic Co-operation and Development (OECD) test guideline no. 423 (https://doi.org/10.1787/9789264071001-en). Briefly, 12 mice were taken and divided into two groups comprising 6 mice in each group. Animals were acclimatized for 7days prior to experimentation. Group 1 mice were given sterile water and group 2 mice were given ApEtOH (2000 mg kg^{-1} body weight single dose). Animals were checked for mortality and morbidity signs throughout the experimental period. On 7th day, mice were euthanized and blood was collected for haematological parameters and serum was isolated to study biochemical parameters (Table 3). Change in body weight, weights of vital organs like liver, spleen, kidney, heart, and lungs were also recorded (Figure 5).

Table 3: Effect of ApEtOH as a single acute oral dose on haemogram and serum biochemical parameters.

Parameters	CONTROL	ApEtOH 2000 mg kg^{-1}
Body weight (g)	26.72 ± 0.58	29.50 ± 1.66
Haemoglobin (g/dL)	16.28 ± 0.43	17.03 ± 0.21
RBC (million/mm^3)	6.82 ± 0.71	6.61 ± 0.24
WBC (*1000/mm^3)	6.90 ± 0.71	6.95 ± 0.25
SGOT (U/L)	26.56 ± 2.31	29.08 ± 2.17
SGPT (U/L)	23.85 ± 1.68	25.40 ± 2.46
Creatinine (mg/dL)	1.85 ± 0.03	1.73 ± 0.04
Triglyceride (mg/dL)	153.64 ± 9.88	167.08 ± 5.42
Cholesterol (mg/dL)	154.47 ± 4.49	145.53 ± 5.38
Bilirubin (mg/dL)	0.42 ± 0.05	0.39 ± 0.04

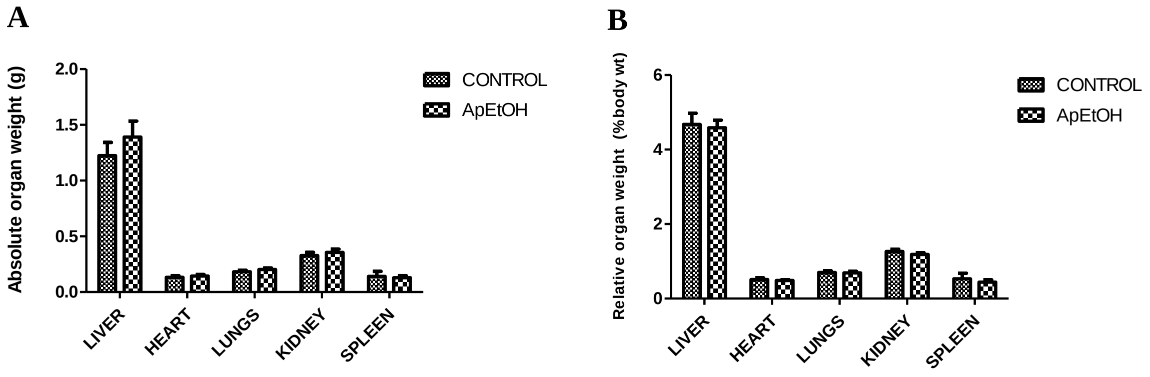

FIGURE 5: Effect of ApEtOH on absolute (A) and relative organ weight (B) in acute oral toxicity studies

Sub-Acute oral toxicity of ApEtOH: Sub-acute oral toxicity of ApEtOH was conducted according to the Organization for Economic Co-operation and Development (OECD) test guideline no. 407 (https://doi.org/10.1787/9789264070684-en). Briefly, 30 mice were taken and divided into four groups comprising 6 mice in each group. Animals were acclimatized for 7days prior to experimentation. Group 1 mice were given 0.6% CMC and mice in group 2, 3, 4 and 5 were given different concentrations (5, 50, 300,750 mg kg^{-1} body weight) of ApEtOH (7 days a week for 28 days). Animals were checked for mortality and morbidity signs throughout the experimental period. On 28th day, mice were euthanized and blood was collected for haematological parameters and serum was isolated to study biochemical parameters (Table 4). Change in body weight, weights of vital organs like liver, spleen, kidney, heart, and lungs were also recorded (Figure 6).

Table 4: Effect of ApEtOH as sub-acute oral dose (5, 50, 300 and 750 mg kg^{-1}) on haemogram and serum biochemical parameters.

Parameters	CONTROL	5 mg kg^{-1}	50 mg kg^{-1}	300 mg kg^{-1}	750 mg kg^{-1}
Body weight (g)	31.98 ± 0.54	32.04 ± 1.93	31.40 ± 0.77	30.80 ± 1.72	33.63 ± 1.25
Haemoglobin (g/dL)	12.70 ± 0.62	13.25 ± 1.10	13.74 ± 0.33	14.05 ± 0.49	13.83 ± 0.41
RBC (million/mm^3)	7.10 ± 0.39	7.61 ± 0.20	7.85 ± 0.15	7.64 ± 0.14	7.71 ± 0.09
WBC (*1000/mm^3)	8.48 ± 1.01	8.45 ± 0.42	10.09 ± 0.93	9.59 ± 0.58	9.78 ± 0.44
SGOT (U/L)	22.98 ± 1.44	25.01 ± 0.42	26.95 ± 2.63	22.49 ± 0.93	25.98 ± 0.51
SGPT (U/L)	25.98 ± 1.48	29.32 ± 0.86	31.51 ± 1.62	31.89 ± 1.17	28.79 ± 1.32
Creatinine (mg/dL)	1.57 ± 0.29	1.84 ± 0.06	1.89 ± 0.05	1.96 ± 0.06	1.91± 0.03
Triglyceride (mg/dL)	150.34 ± 11.01	134.16 ± 8.04	118.38 ± 7.94	133.74 ± 4.65	121.40 ± 2.73
Cholesterol (mg/dL)	153.61 ± 2.12	142.44 ± 2.05	144.44 ± 2.27	155.01 ± 3.31	147.02 ± 2.35
Bilirubin (mg/dL)	0.45 ± 0.03	0.44 ± 0.03	0.49 ± 0.04	0.49 ± 0.02	0.44 ± 0.03

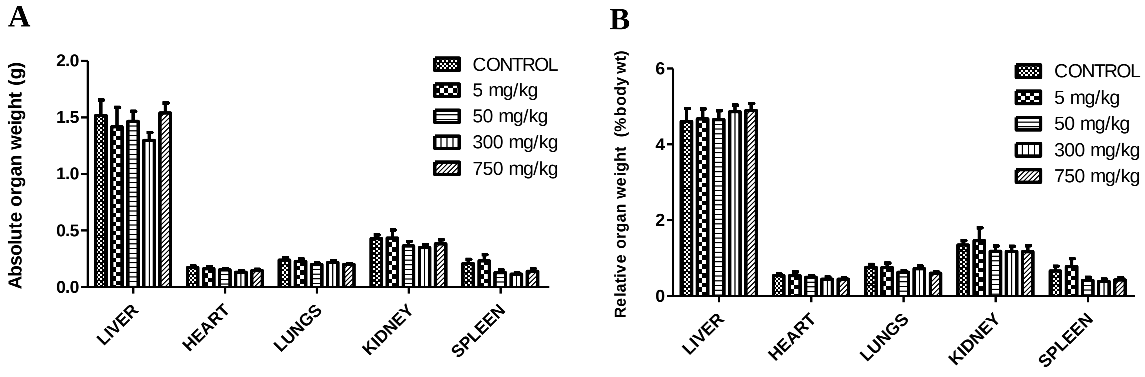

FIGURE 6: Effect of ApEtOH on absolute (A) and relative organ weight (B) in sub-acute oral toxicity studies
